# Terahertz time-gated spectral imaging for content extraction through layered structures

**DOI:** 10.1038/ncomms12665

**Published:** 2016-09-09

**Authors:** Albert Redo-Sanchez, Barmak Heshmat, Alireza Aghasi, Salman Naqvi, Mingjie Zhang, Justin Romberg, Ramesh Raskar

**Affiliations:** 1 Department of Media arts and Sciences, MIT Media Lab, Massachusetts Institute of Technology, Cambridge, Massachusetts 02139, USA; 2 Department of Electrical and Computer Engineering, Georgia Institute of Technology, Atlanta, Georgia 30332, USA; 3 Department of Automation, Tsinghua University, Beijing 100084, China

## Abstract

Spatial resolution, spectral contrast and occlusion are three major bottlenecks for non-invasive inspection of complex samples with current imaging technologies. We exploit the sub-picosecond time resolution along with spectral resolution provided by terahertz time-domain spectroscopy to computationally extract occluding content from layers whose thicknesses are wavelength comparable. The method uses the statistics of the reflected terahertz electric field at subwavelength gaps to lock into each layer position and then uses a time-gated spectral kurtosis to tune to highest spectral contrast of the content on that specific layer. To demonstrate, occluding textual content was successfully extracted from a packed stack of paper pages down to nine pages without human supervision. The method provides over an order of magnitude enhancement in the signal contrast and can impact inspection of structural defects in wooden objects, plastic components, composites, drugs and especially cultural artefacts with subwavelength or wavelength comparable layers.

## Abstract (web summary)

Terahertz radiation may be used to nondestructively detect and study defects and structures within materials. Here the authors use terahertz time-gated spectral imaging to extract occluded text from paper pages with subwavelength spacing.

## Graphical abstract

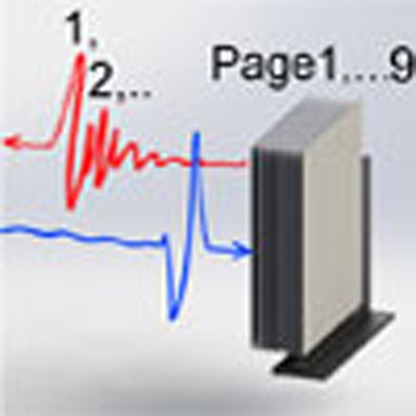

Terahertz time-domain spectroscopy (THz-TDS) is a leading method for spectroscopy, imaging and nondestructive testing^1^,^2^ in the frequency range of 0.1–10 THz (refs 3, 4). The method can detect structural defects in foams, wooden objects^5^, plastic components^6^, composites^7^, pharmaceutical products' coatings^8^ and cultural artefacts^9^,^10^,^11^. In contrast to infrared-based time-of-flight cameras, optical coherent tomographic techniques^12^,^13^,^14^ and X-ray techniques, THz-TDS provides both fine time resolution and broadband spectral signatures for a variety of dielectric materials. These advantages have motivated researchers to use computational techniques^15^,^16^ to empower the yet-maturing THz hardware^3^.

Despite the prevalence of sub-millimetre layered structures in industry, biology and objects of cultural value^5^,^6^,^7^,^8^,^9^,^10^,^11^, conventional THz-TDS is incapable of deep content extraction for three well-known reasons^8^,^9^,^10^,^11^: signal-to-noise ratio (SNR) drops with depth (or increasing number of layers), the contrast of the content is much lower than the contrast between dielectric layers, the content from deeper layers are occluded by the content from front layers. Here we introduce a time-gated spectral imaging technique that overcomes all of these challenges to extract occluding content from layers whose thicknesses and separations are comparable to the wavelength. The method uses the statistics of the THz electric field (E-field) to lock into each layer position and then uses a time-gated spectral kurtosis averaging to tune to the spectral images with the highest contrast on that layer. This provides layer extraction at low SNR<10 dB and intensity images with up to 18 times higher contrast compared with simple amplitude mapping. The extraction overcomes partial occlusion by a shape composition algorithm^17^. To demonstrate, occluding textual content was extracted from a sample similar to a closed book with single-sided pages down to nine pages without human supervision. The proposed method provides a capability to inspect densely layered samples prevalent in industry (for example, coatings and polymer-based laminates), geology and specifically objects of cultural value (for example, documents and art works). The study also thoroughly discusses the advantages and limitations of this technique.

## Results

### Experimental set-up specifications and challenges

**Figure 1:**
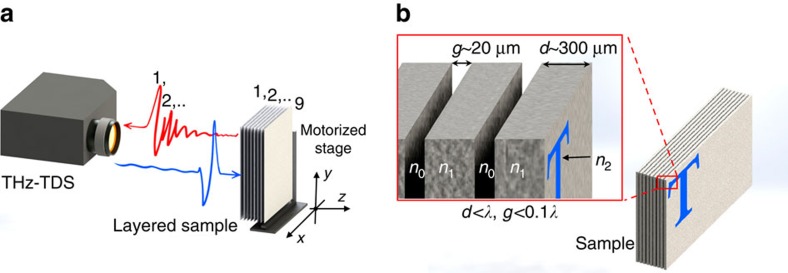
Measurement geometry and sample schematics. (**a**) Confocal THz time-domain (THz-TDS) measurement is used in reflection geometry. *x–y–z* are the axis of a Cartesian coordinate system that is kept consistent throughout this study. The 9-page sample is held on an *x–y*-motorized stage that enables mechanical raster scanning in *x–y* plane. A THz pulse is transmitted. The electric field is a bipolar pulse as shown schematically in blue. The reflected signal (shown in red) has a series of dense reflections (usually more than nine) from the layered sample that provides time-of-flight information for boundaries of pages in *z*. (**b**) The layered sample is composed of nine packed paper layers. Each layer is 300 μm thick and the non-uniform gaps between the layers are ∼20 μm after pressing the pages together.

The experimental set-up is in confocal geometry to raster the sample (Fig. 1a). For each spatial point, a THz time-domain measurement records the reflections of the E-field from the layered sample with 40 fs time resolution. The sample consists of nine layers of paper with a single character written on each page. The pages are 300 μm thick and are pressed together to mimic the structure of a closed book (Fig. 1b). The pages have text only on single side and are thicker than standard letter-sized paper. Reflection geometry provides time-of-flight information for each reflection that is generated from each layer (Fig. 1a; Supplementary Fig. 1). Therefore, indexing the pulses in time will provide a direct measurement of the position of the different layers within a sample.

**Figure 2:**
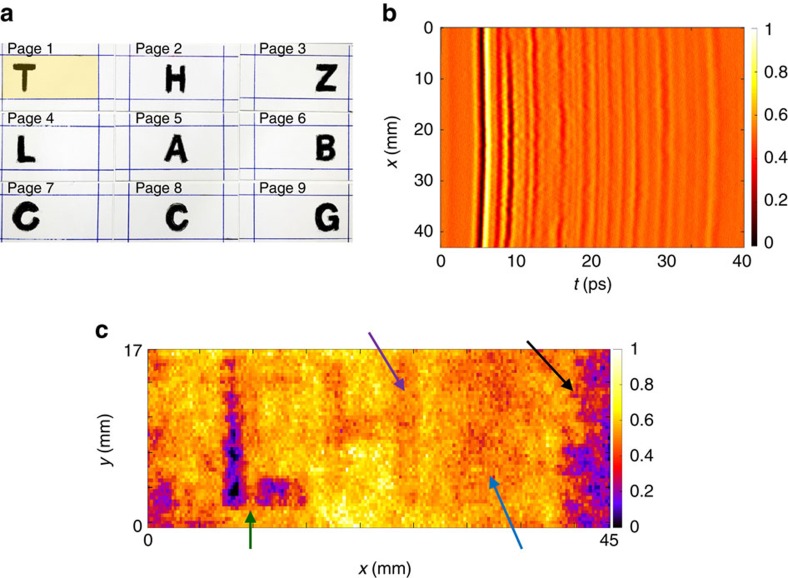
Sample description and raw measurements overview. (**a**) Nine roman letters T, H, Z, L, A, B, C, C and G are written on nine pages. The pages are then stacked on top of each other, respectively. The orange highlighted area is the scanned area facing the system. (**b**) THz-TD image obtained in *x–t* or equivalently *x–z*. Image intensity indicates normalized field amplitude in arbitrary units. The values below 0.5 indicate negative field amplitude. Pulse echoes indicate the depth of each page. (**c**) A time frame of the recorded electric field amplitude data cube. The scale bar indicates the normalized field amplitude in arbitrary unites. Arrows show the effect of occlusion (green arrow T is occluding L), shadow (purple and blue arrow, shadow of H and Z) and the interlayer reflection-induced noise (black arrow). The latter is comparable to the signal level of the letters. The horizontal and vertical axis are *x* and *y* axis in millimetre.

The layers contain T, H, Z, L, A, B, C, C and G, respectively, from page 1 at the front to page 9 at the back of the stack (Fig. 2a). Despite the pressure, an air gap is forced due to the roughness of the pages (the paper layers are common wood-based sketching pages with no specific surface finish). Surprisingly, this gap of ∼20 μm shows up as a single peak in the measurements and can be exploited to detect layers despite being an order of magnitude smaller than the illumination wavelength. Figure 2b shows a cross-section in which we can see the reflections from the different layers, including the front and the back of each page. Figure 2c shows a time instance *t* of the recorded (*x–y–t*) data cube. As in Fig. 2c, there are four major sources of ambiguity in the measured data: shadows of the superficial pages, occlusion from superficial characters, general system noise and finally the interferometric noise induced by inter-reflections, roughness and slight curvatures of the layers. For example, the shadowing effect of characters H (page 2) and Z (page 3), and occlusion of character T (page 1) on the L (page 4) can be seen in Fig. 2c.

### Content extraction procedure

Page position extraction from a *y–t* cross-section (Fig. 2b) using conventional deconvolution methods, such as CLEAN or frequency-domain deconvolution, fails beyond page 5 because of pulse dispersion and low SNR (CLEAN is not an acronym and it refers to the word-clean beam-in radio astronomy where this algorithm was initially developed), while edge detection methods can suffer from sensitivity to noise. On the other hand, purely wavelet-based peak finding methods can suffer from pulse distortion and overlap. This motivates the development of our Probabilistic Pulse Extraction (PPEX) algorithm, which exploits the statistics specific to THz time-domain pulses. PPEX calculates the probability of each point of being an extremal value in the waveform based on the amplitude, first derivative (for example, velocity) and the statistical characteristics of the noise in the waveform (Supplementary Notes 1 and 2). PPEX first computes an energy value based on the amplitude and the velocity extracted from the waveform, with high energy corresponding to large amplitudes and low velocities (Supplementary Fig. 2a). This corresponds to candidate points being extremal. This provides a different filtering mechanism to separate the signal from noise (Supplementary Notes 3–5). PPEX then uses the histograms of the amplitude and velocities to define a probability distribution for both amplitude and velocity (Supplementary Fig. 2b–e). On the basis of such probability distribution, PPEX computes a combined probability of a point to be extremal. However, PPEX does not identify which candidates correspond to a certain peak. Experimental and simulation results indicate that candidates tend to the group around a real peak of the pulse. We use k-means clustering to match the candidates to different peaks. For each cluster, the representative value can be selected by taking either the candidate with the highest amplitude or averaging the positions within a cluster (Supplementary Fig. 2e).

The refractive indices of the paper materials and phase shift at the interfaces can scale the time axis for depth extraction. However, since the air gap refractive index and the material refractive index are known (or are measurable with the same system), the depth of each layer is recoverable from the known scan range. Also, in case of unwanted ambiguity in the exact thickness of the paper, the performance of the algorithm in finding the layers is yet left intact.

Unfortunately, even when the layer positions are extracted correctly, the amplitude contrast between peak reflected E-field from blank paper and paper with a character is lower than interlayer reflection noise caused by larger refractive index difference between air and paper. This means that the inter-reflection noise of deeper layers will completely overshadow the original signal contrast from the characters. The contrast also degrades quickly with depth as the SNR decreases. Therefore, a method that fine tunes to the highest spectral contrast, such as the proposed time-gated spectral imaging, is absolutely necessary to retrieve the content buried in heavy noise.

There are three reasons for such poor contrast: the first reason is the low contrast in the complex refractive index of the ink material and the paper, certainly an ink or printing material that has a larger reflection contrast with the paper material in THz range would enhance the results; the second reason is the extremely small thickness of the ink layer on the paper that reduces the overall absorption contrast. It is essential to work with such low thicknesses of the ink materials to demonstrate our technique in a realistic scenario where letters are naturally handwritten on normal paper pages. The third reason for poor contrast on the pages is lower SNR level that is present at each individual frequency frame. This low SNR is the direct result of contribution of other pages spectra to a general Fourier transform of the data and also the spreading of contrasting the frequencies across the frames.

The time-gated spectral imaging uses a thin time slice (∼3 ps) of the waveform around the position of the layers extracted by PPEX to compute the Fourier transform. This operation unavoidably introduces some higher-frequency artefacts, but as a tradeoff the gain in the contrast is significantly better (over an order of magnitude) than a simple amplitude mapping of the waveform. Furthermore, the kurtosis of the histograms of the resulting images in the frequency domain provides a mechanism to select the frames with the highest contrast between the paper and the content material. The kurtosis is induced by the presence of two distinct reflective materials on each layer: the higher the contrast between blank paper and paper with ink in a certain frequency, the higher the kurtosis (details of the time-gated Fourier analysis at Supplementary Note 6). In essence, our technique increases the contrast by addressing both of these issues; it first windows the data in time to filter out the unwanted contribution from other pages and it then aggregates the signal power by tuning into frequency frames that provide the highest contrast between the two materials. The dominant contrasting components for our experiment were mostly present in the 500–600 GHz frequency range with few frames outside of this range. This range slightly varies based on the type of paper and ink, but it is mostly consistent throughout different pages of the same stack. It is noteworthy that the lower spatial resolution of lower frequency range and also higher noise level of hyper terahertz range can also contribute to lower kurtosis value along with the material absorption spectrum itself. In fact, these factors gradually mask (reduce) the contrast as we move towards the lowest and highest end of the THz pulse spectrum. However, these contrast reducing factors seem to be negligible compared with material spectral contrast in the 300 GHz to 1.5 THz range.

**Figure 3:**
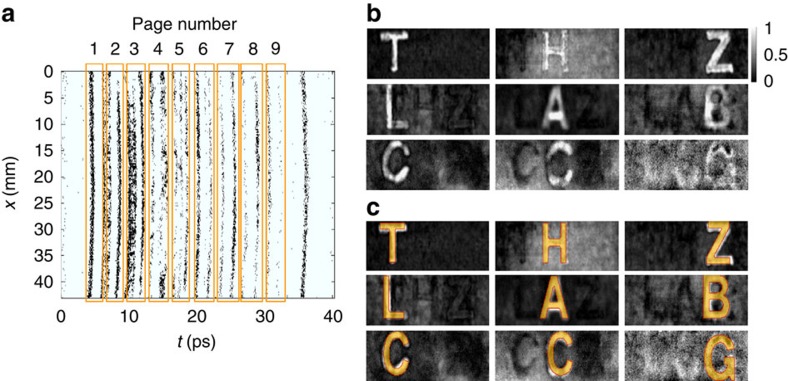
Unsupervised content extraction with THz time-gated spectral imaging. (**a**) Layers are identified in time based on the statistics of the reflected bipolar THz field signal. Image is binary. (**b**) The technique uses kurtosis of the time-gated Fourier transform to contrast the content. Grey scale colour bar indicates the normalized amplitude of the averaged frequency components in arbitrary units that is output from the contrast enhancement procedure. Each page is normalized separately, and horizontal and vertical axes are omitted for simplicity. (**c**) Convex cardinal shape composition (CCSC) algorithm extracts the occluded characters through THz noise down to page 9. The detected letters are highlighted with light orange.

The results for the first three layers are easily recognizable by the human eye (Fig. 3b). As the depth increases, the intensity of the characters becomes significantly non-uniform, and the interference between layers becomes significant—classifying the eighth and ninth layers, for example, is a challenging character recognition problem. The convex cardinal shape composition algorithm (CCSC) automatically matches regions of relatively high intensity against combinations of shapes (letter templates) at different locations and orientations. This is formulated as an optimization problem; each possible combination of shapes has an associated energy that scores its match to the image, and the algorithm searches over all such possibilities to maximize this score. This search is made computationally tractable by relaxing the combinatorial search into a convex program that can be solved with standard optimization software. Figure 3c shows that CCSC is successful despite the presence of significant shadowing and occlusions in the deeper layers. The CCSC optimization is detailed in Supplementary Note 7.

### Performance evaluation

**Figure 4:**
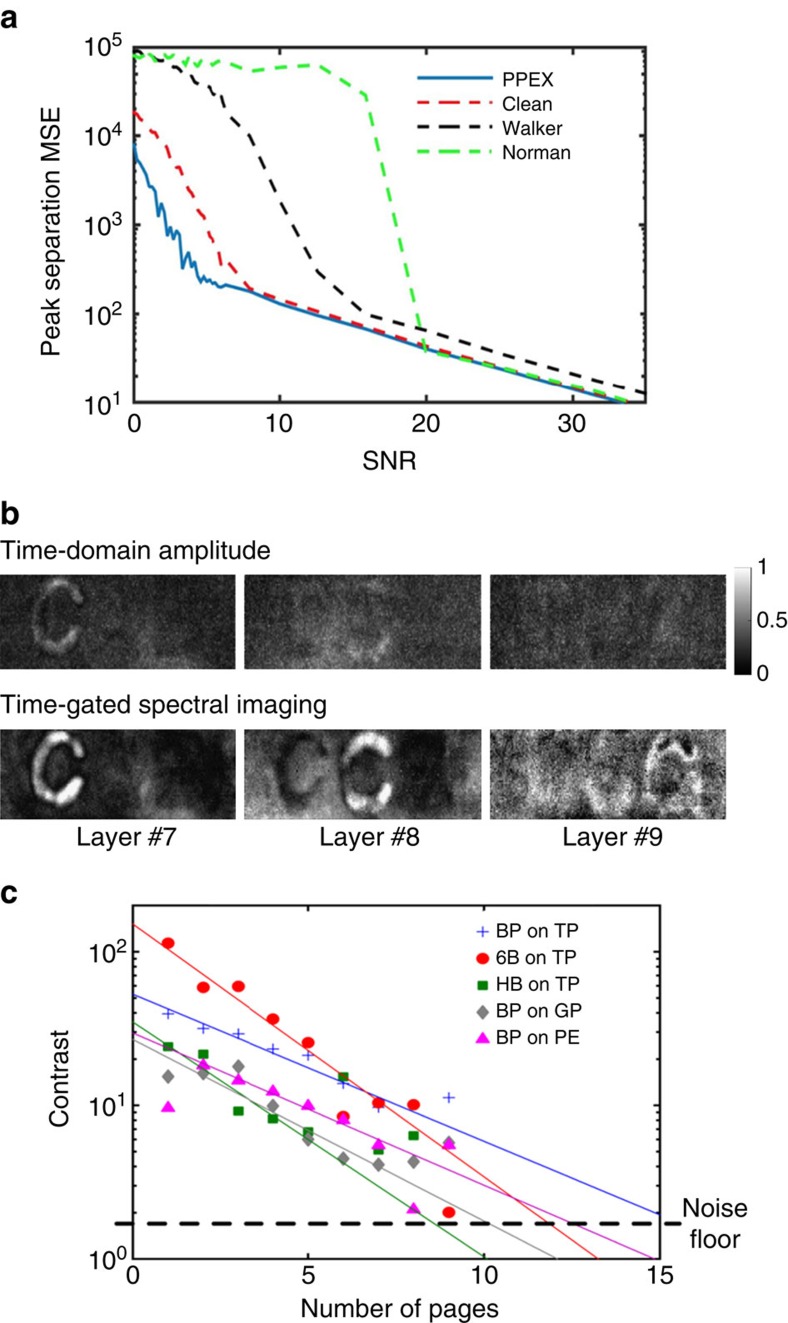
Procedure performance highlights. (**a**) PPEX exploits THz wave statistics and outperforms the conventional CLEAN (dashed red), Walker (dashed black) and Norman (dashed green) deconvolution at the low SNRs (<10 dB) typically encountered in THz depth sensing. (**b**) Time-domain amplitude versus time-gated Fourier transform. Contrast is enhanced by tuning to spectral differences between two materials. Each image is normalized separately. The grey scale colour bar indicates the normalized field amplitude at time domain and average of contrasting Fourier components amplitude in time-gated spectral imaging. All image intensities are in arbitrary units. The text is written with pencil. (**c**) Reduction in contrast level for different writing utensil and paper material combinations. BP stands for blue pen ink; 6B and HB are two types of graphite-based pencil; TP stands for typical wood-based paper; GP stands for glossy paper; PE stands for high-density polyethylene. On the basis of fitting curves, contrast level drops down exponentially with depth. The horizontal dashed line shows the noise floor for our THz-TDS system.

The proposed three steps work together to extract contents as deep as possible (Supplementary Movie). Figure 4a shows the superior performance (about an order of magnitude at SNR<10 dB—SNR is 20log(|*E*_s_|.|*E*_n_|^−1^)) of PPEX compared with a set of standard deconvolution techniques (Supplementary Fig. 3). *E*_s_ is the signal component of the measured electric field amplitude and *E*_n_ is the noise component. Detailed comparison with edge detection techniques and CLEAN deconvolution is shown in Supplementary Figs 4a–e and 5a–d Low-pass filter was applied to the data before feeding it to the deconvolution for Fig. 4a comparison. The induced dispersive behaviour in the time domain is not because of the dispersion in the bulk of the material itself as in the case of optical waveguides. Rather it is a dispersive behaviour induced by the frequency-dependent response of the subwavelength air gaps. The gaps respond mostly to higher frequencies and are rendered transparent in lower frequencies. This dispersive response of the layered structure encourages the use of algorithms that consider temporal statistics rather than varying frequency components. In addition, PPEX does not require the use of a reference pulse; therefore, is far less affected by the dispersion of the pulses than deconvolution methods, which require the measurement or modelling of an ideal pulse (a full performance analysis is in Supplementary Note 3). For example, wavelet transforms have been used in decomposing and denoising of THz signals^18^,^19^,^20^,^21^,^22^,^23^,^24^. Sole wavelet-based peak extraction methods provide inaccurate results for peak finding in THz signal from dense-layered structures (Supplementary Fig. 6a–d). This is because of varying peak width in the reflected signal that projects each peak into a different wavelet scale causing inaccuracy in localization. Also, the overlap of the peaks caused by subwavelength structure misguides the transform to false detections (Supplementary Note 4).

If PPEX was to use only the amplitude and not the velocity of the waveform, the algorithm would have been more susceptible to water vapour-induced oscillations in the time domain^25^. However, the velocity and overall energy of these peaks are usually much lower than the original signal that is reflected from solid–air interface and this helps PPEX eliminate these peaks in the detection process. Water vapour-induced ripples in the electric field can be partially compensated for deconvolution methods as well, but the strong reference dependency of these techniques means that any change in the humidity level can negatively impact the deconvolution.

Figure 4b shows the difference between amplitude mapping in the time domain and the time-gated Fourier transform. The time-gated spectral analysis based on kurtosis provides up to 18 times more contrast for the eighth layer. For the ninth layer, the signal level is too low to accurately estimate the contrast improvement (our estimation is ∼10.5). However, in Fig. 4b, we see that the character is now completely recognizable to both the human eye and the CCSC algorithm.

The signal loss with depth is a major burden on reading deeper layers. This loss is caused by consecutive reflections at the material interfaces (both the back and the front of each layer) and also by the exponential Lambertian absorption of the layers themselves. The reflected signal level is not the bottleneck to content extraction at deeper layers (we can detect 15 pages with the first step of time-gated spectral imaging; PPEX), it is rather the contrast between the signal levels of the printed characters and blank paper that limits the extraction to nine pages. Therefore, based on the THz contrast between the layer and the content at that layer the depth of extraction can vary. Figure 4c shows the experimental measurements of the reflection contrast for different materials combinations (more details in Supplementary Note 8 and Supplementary Table 1). The lines are an exponential interpolation of the measured data set. The figure provides an estimation of the number of pages that could be read given an input THz SNR, Lambertian absorption of the pages and cascaded Fresnel reflections at each layer. The noise floor line is the ultimate system noise level. As in Fig. 4c, the contrast values below 10 are very noisy due to inter-reflections and other noise sources.

Since THz waves are zero mean oscillatory signals and wavelet basis can be generated to closely resemble such signals, there are numerous studies pioneered by Mittleman *et al.* and MacPherson *et al.* that promote decomposition and processing of THz waves in different wavelet spaces. For example, wavelet has been used for low-frequency background denoising in Fourier deconvolution^18^,^19^,^22^,^24^, efficient tomographic reconstruction^20^, spectral contrasting of different materials^21^ and compressive acquisitions of THz images^23^. Wavelet basis also have been proposed for deconvolution of THz pulses in time^26^,^27^. We have applied and compared the performance of different wavelet-based deconvolution schemes in the Supplementary Note 5; the comparison of PPEX, deconvolution using Frequency-Wavelet Domain Deconvolution^26^, wavelet-based time-domain deconvolution using Tikhonov regularization and 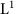 regularization shows that the performance of PPEX is yet superior in deeper layers (Supplementary Figs 7a–e and 8a–f).

This may be counter intuitive, since wavelet is considered to be in direct correspondence with THz signals and that is why wavelet decompositions have an edge over variety of techniques. However, such high sensitivity is not directly beneficial in our application. Because of such sensitivity, the results will suffer notably from even minor dispersions in the reflected pulses (Supplementary Note 5). This is not the case for PPEX, as it tunes into dominant peaks in reflected energy by rejecting the lower-energy oscillations through energy histogram thresholding. In addition, similar to any other deconvolution, wavelet-based deconvolution methods require reference measurement that is not needed for PPEX.

## Discussion

The proposed framework has several advantages over conventional windowed techniques^28^. First the window size itself is fed from PPEX layer extraction, which assists in screening the contributions from individual layers with minimal distortion from other layers. Second, the use of kurtosis as a measure of image contrast is a low complexity (computationally fast) and reliable way of identifying the higher-contrast images. Application of kurtosis suits our problem very well, since we are analysing almost-binary images, and kurtosis would be an optimal tool to evaluate the peakedness or binary nature of the images (Supplementary Fig. 9a–d). Last but not the least, the kurtosis processing is a matched pre-processing step for the CCSC step of noisy images (see examples in reference ref. 17)

While PPEX does not require reference signal, it only finds the most probable energy peaks that are to represent a dominant reflection from a boundary. Due to oscillatory nature of THz field, there can be conditions (high humidity or very thin nearby layers) where the oscillations in the tail of the bipolar E-field are so large that they generate comparable dominant peaks in the calculated energy value. This can potentially cause error in the layer detection process and limit the application of PPEX in such unlikely conditions. However, as fully discussed in Supplementary Note 1, the amplitudes of consecutive (higher order) reflections from the inter-reflections decays exponentially and peaks from water absorption are usually orders of magnitude smaller than the dominant peaks induced by air–solid interface.

Another potential limiting factor can be the dispersion of the layers, especially a stack of layers with high refractive indices and thickness comparable to wavelength of the pulse can act as a strong frequency-dependent reflector that may limit the method for certain materials and geometries. The 300 μm paper used in this study is thicker than a normal (A4 or letter size) 100–250 μm paper thickness, however, as detailed in Supplementary Note 8 the dominant bottleneck is not thickness of the paper but rather the contrast of the ink with that paper material in the THz frequency range. Thicker papers were easier to use in this study, since they do not warp during preparation. The warping of the pages (if significant enough) can cause PPEX to fail, since it can push the content of one time gate to another at different *x–y* positions, warping can also create raster scan sweeping distortions. Removing such distortions from significantly warped layers can be a topic of future study.

In case these types of error are output from PPEX, they would still not directly propagate to the incorrect content extraction from pages, they would only create ambiguity about the page number, convex cardinal indeed can tolerate overlapped and occluded content that may result from such errors (Supplementary Figs 10 and 11). Finally, the depth resolution is fundamentally limited by half the coherency length of the THz wave. Our study was also limited to pages with content on one side. Double-sided pages with contents on both sides would have forced further sophistication of our methodology (Supplementary Fig. 12).

Although there have been few attempts to fabricate compressive THz cameras^15^,^16^,^29^, the high-resolution THz-TDS used in this study is currently available only in the raster scanning mode. This limits the applications that are time sensitive on the acquisition end. Fortunately, THz-TDS power and sensitivity levels have been constantly rising in recent years^30^,^31^. This is encouraging as the number of layers readable to our method increases with SNR. In addition to the inspection applications mentioned above, our time-gated method allows us to decode structure at deeper layers^32^. This is especially interesting as it allows direct contact of adjacent layers and the use of widely available paper and carbon-based ink. While similar in principle, there are major differences between THz-TDS and ground-penetrating radar that are detailed in Supplementary Table 2.

Finally, our demonstration of content extraction from densely packed layers points to the level of practicality that computational THz-TDS can offer. The proposed time-gated spectral imaging is not limited to THz-TDS and can be of significant benefit to other time-resolved techniques. We find that the method is limited by the contrast level between the signal from content and blank portion of each layer rather than reflection signal *per se*. The work thus provides fundamental insight into three-dimensional spectroscopy and inspection of densely layered structures with sub-millimetre thicknesses.

## Methods

### System specification

The data were acquired with a FICO THz time-domain system manufactured by Zomega Terahertz Corporation. The system has a bandwidth of 2 THz and time delay range of 100 ps. The FICO generates THz pulses via a photoconductive antenna pumped by a femtosecond laser. Electro-optic sampling and balanced detection are used for THz detection. A pump-probe approach with a mechanical delay allows recording the shape of the THz pulse in the time domain (reflected power is in the tens of nW range). The THz beam is focused on the sample with 25.4 mm focal lens and diameter TPX lens (Polymethylpentene lens). The sample was mounted on a *XY*-motorized stage, so that an image can be acquired by raster scanning. Imaging area was 20 × 44 mm and step size was 0.25 mm. The system offers a dynamic range of 65 dB in power.

### Software specifications

Data cube captured by the system were processed using MATLAB software. Processing includes the application of PPEX, k-means clustering and CCSC extraction, which take few seconds to complete. Various numerical schemes, such as the alternating direction method of multipliers^33^,^34^ or projected sub-gradient method^35^, can be employed to address the CCSC convex optimization problem. It is recently shown^36^ that CCSC can be also reformulated as a linear program by increasing the number of optimization variables. Through such reformulation, the problem can be addressed in fractions of a second using a desktop computer.

### Data availability

All the raw data and codes that support the findings of this study are available at https://dx.doi.org/10.6084/m9.figshare.3471434.v1 (ref. 37).

## Additional information

**How to cite this article:** Redo-Sanchez, A. *et al.* Terahertz time-gated spectral imaging for content extraction through layered structures. *Nat. Commun.* 7:12665 doi: 10.1038/ncomms12665 (2016).

## Supplementary Material

Supplementary Information Figures 1-12, Supplementary Tables 1-2, Supplementary Notes 1-8, Supplementary References

Supplementary Movie THz Reading through closed book
