# Supplementary material for: Terahertz time-gated spectral imaging for content extraction through layered structures
Source: Nat Commun. 2016 Sep 9;7:12665. doi: 10.1038/ncomms12665 (PMC5023963; doi:10.1038/ncomms12665)
Supplement: Supplementary Information — Figures 1-12, Supplementary Tables 1-2, Supplementary Notes 1-8, Supplementary References [file ncomms12665-s1.pdf]

Supplementary Figures

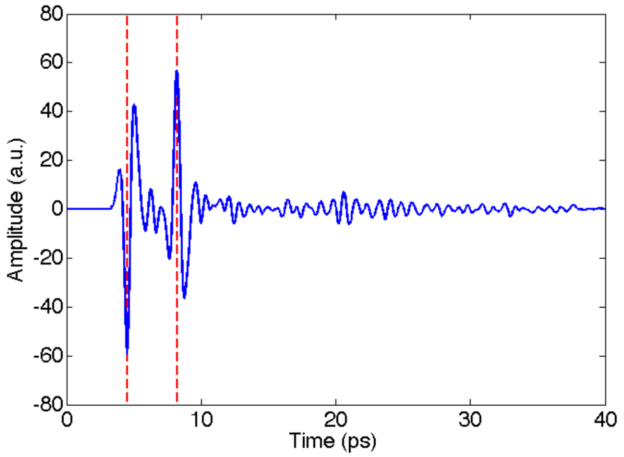

Supplementary figure 1 | The simulated returned signal from a dielectric slab for the reference signal used in the experiments (see Supplementary figure 6a below). The red lines indicate the locations of the dominant impulses, which coincide with the signal dominant peaks.

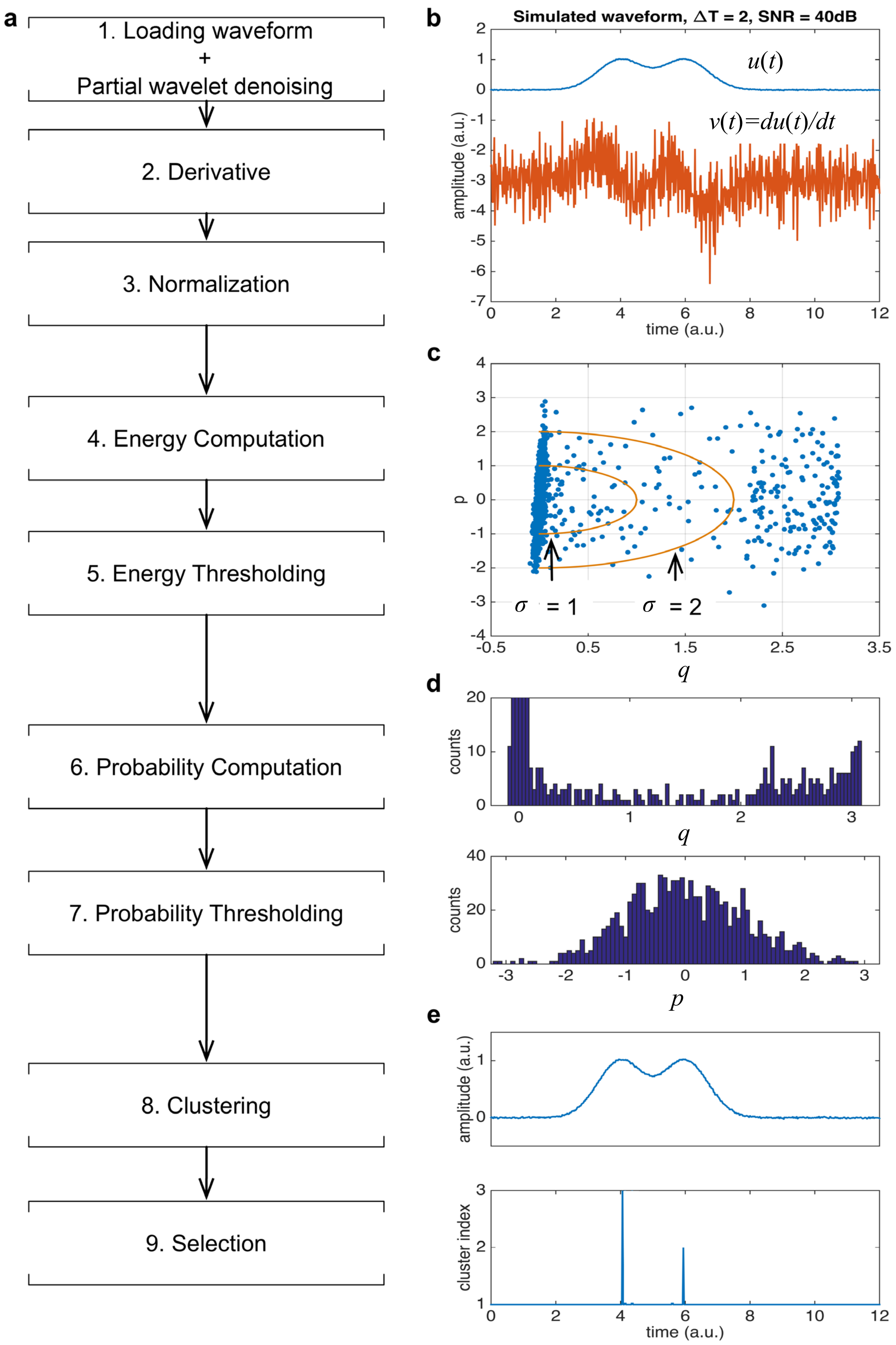

Supplementary figure 2 | PPEX algorithm flow. a, Data pipeline. **b,** An example waveform and its time derivative. **c,** Phase diagram of normalized amplitude and velocity, which allows defining an energy. That energy is used as a first thresholding mechanism to separate noise (low energy) from signal (high energy). **d,** Histograms of amplitudes and velocities to determine the distribution to calculate probabilities. **e,** Peak selection after clustering of candidates.

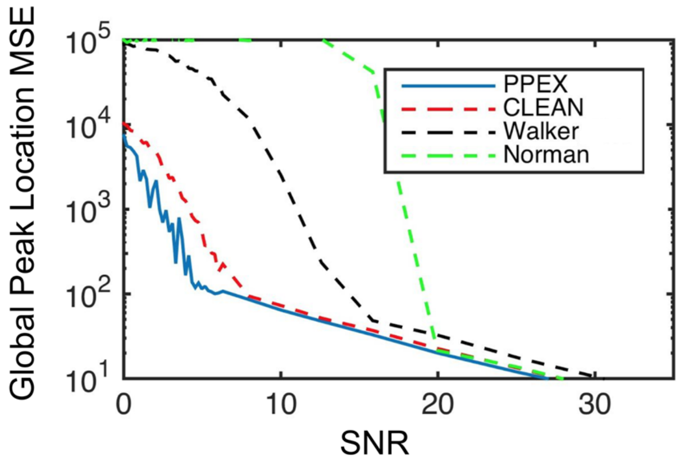

Supplementary figure 3 | Mean Square Error of global peak location of different methods. PPEX is significantly better than frequency based deconvolution reported in Walker and recent robust peak finding method reported in Norman. PPEX also outperforms CLEAN for SNR < 8 dB by an order of magnitude.

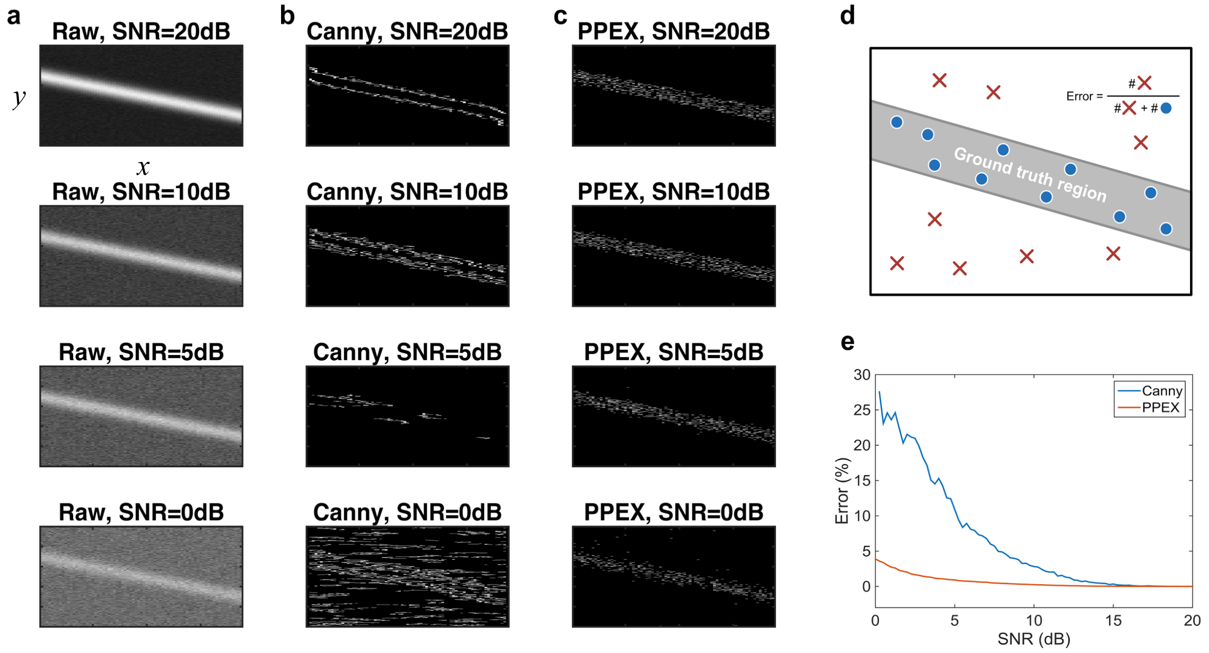

Supplementary figure 4 | Comparison between Canny edge detection and PPEX in finding the position of a simulated peak at different SNR levels. a, Raw simulated data with addition of noise. **b**, Canny results. **c**, PPEX results. **d**, Error calculation scheme. **e**, Error comparison versus SNR. PPEX outperforms Canny edge detection at low SNR encountered in THz depth sensing.

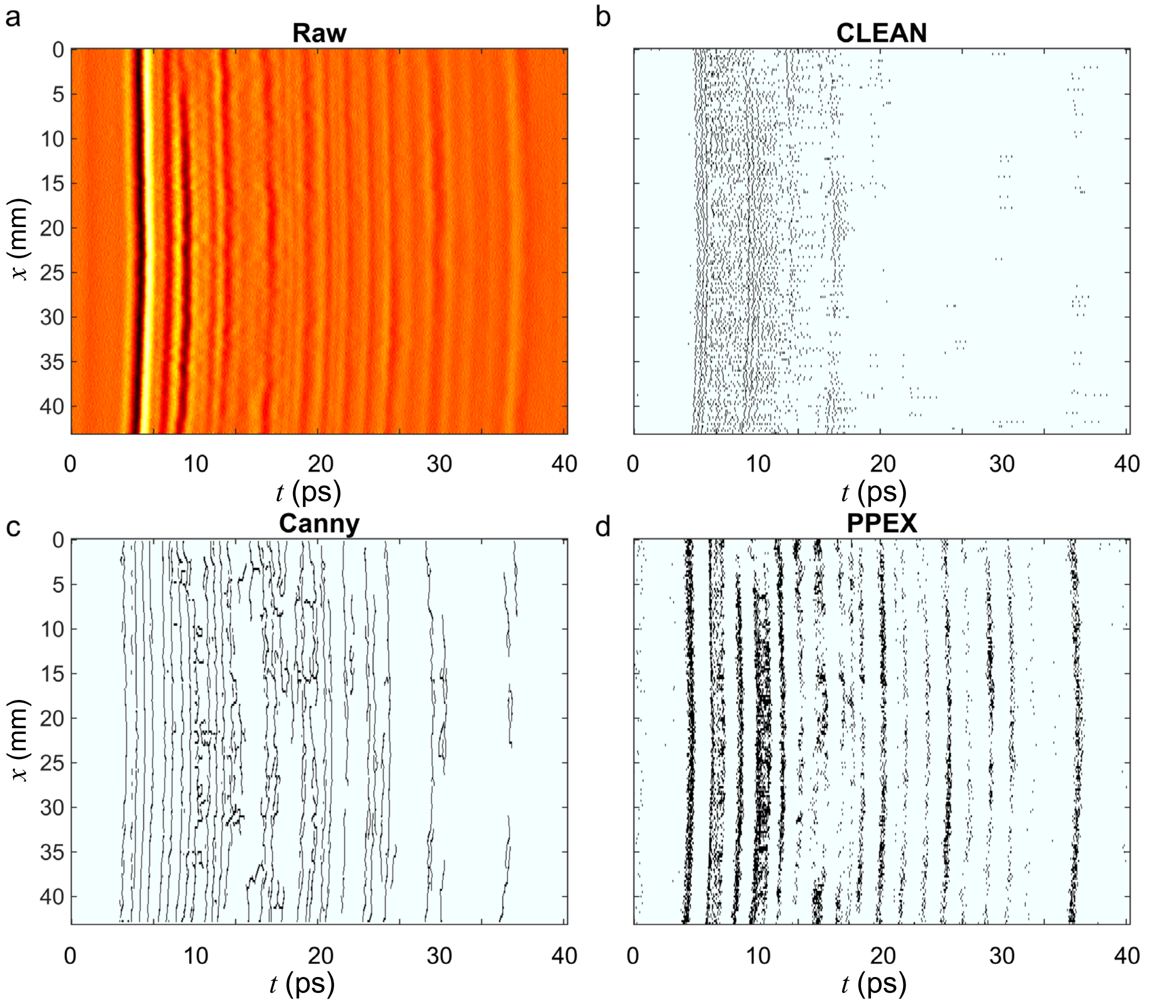

Supplementary figure 5 | Comparison between CLEAN, Canny edge detection and PPEX in finding the position of a layers in experimental data. a, Raw experimental data. **b,** Results from CLEAN deconvolution. **c,** Results from Canny edge detection. **d,** Results from PPEX.

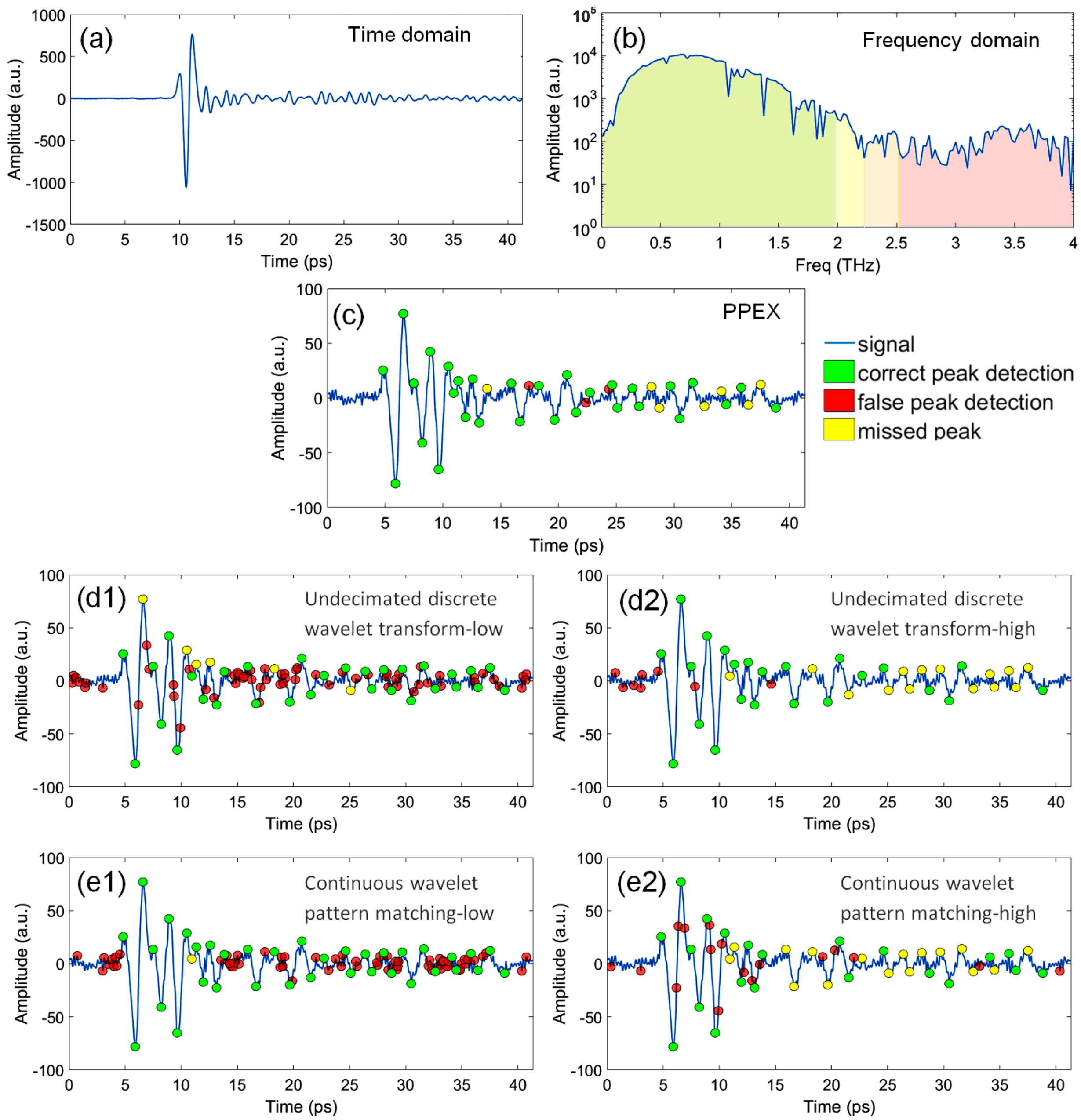

Supplementary figure 6 | Waveform and wavelet applications vs. PPEX. a, Reference waveform. **b,** Reference spectrum. **c,** Peak finding results using PPEX: green, red and yellow respectively correspond to correct detection, false detection and misidentification. **d1,** Application of method in[1] with the same level of smoothing as PPEX. **d2,** Application of method in[1] using a higher level of smoothing. **e1,** Application of method in[2] with low thresholding. **e2,** Application of method in[2] with higher thresholding.

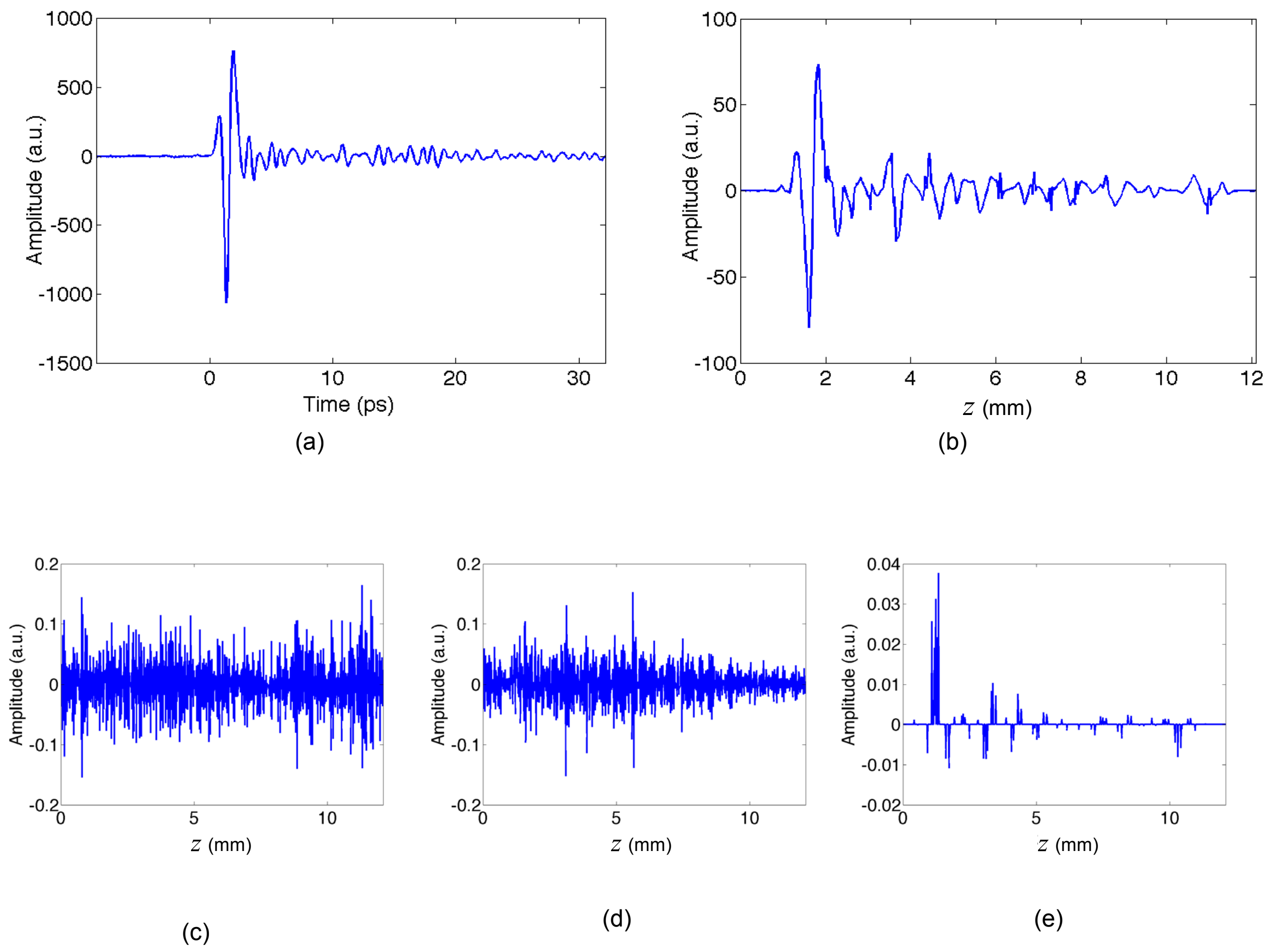

Supplementary figure 7 | The performance of different wavelet-based deconvolution schemes. a, Reference pulse. **b,** The measured reflection from multilayered sample along a single pixel. **c,** Deconvolution of signal in b using FWDD. **d,** Time domain deconvolution using Tikhonov regularization. **e,** Time domain deconvolution using ℓ_1 regularization.

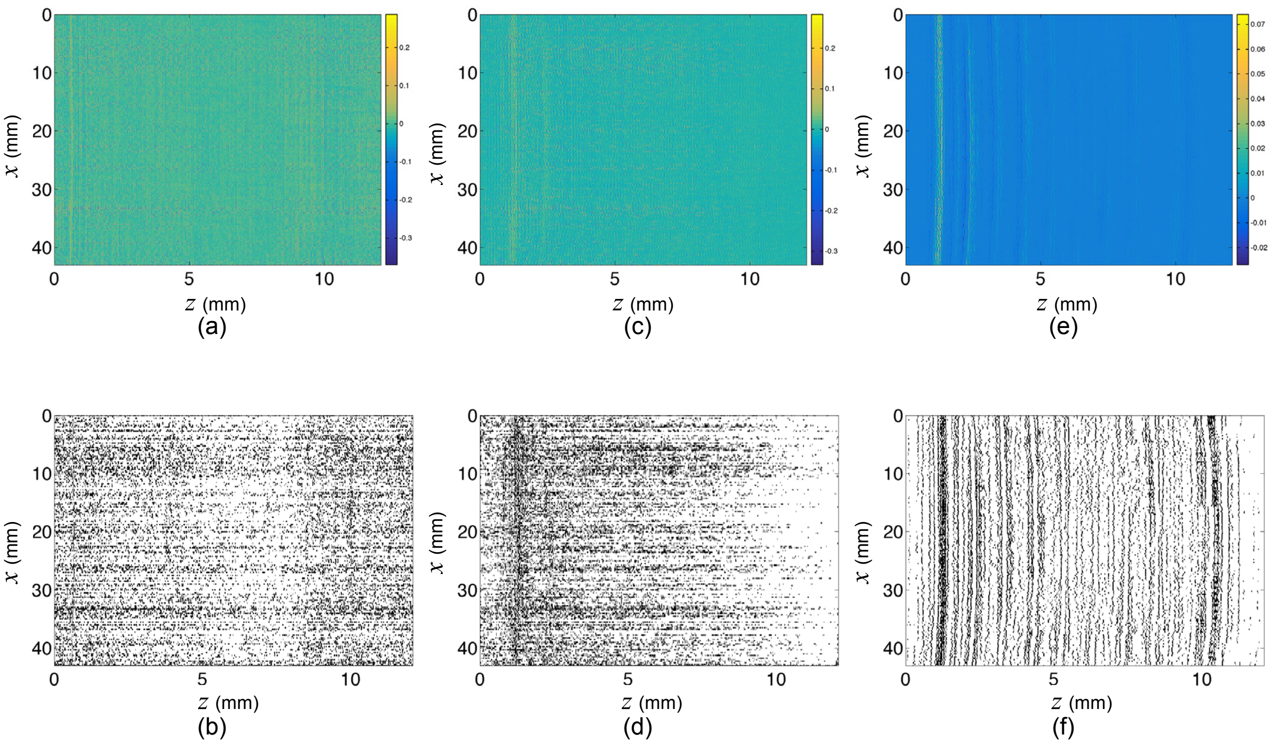

Supplementary figure 8 | Deconvolution of the raw experimental data. a, Deconvolution using FWDD. (The color bar is the deconvolved signal amplitude) **b,** A binary version of the FWDD result in panel a. **c,d,** Recovered image and binary version using time domain deconvolution with Tikhonov penalty. **e,f,** Recovered image and binary version using time domain deconvolution with ℓ_1 penalty.

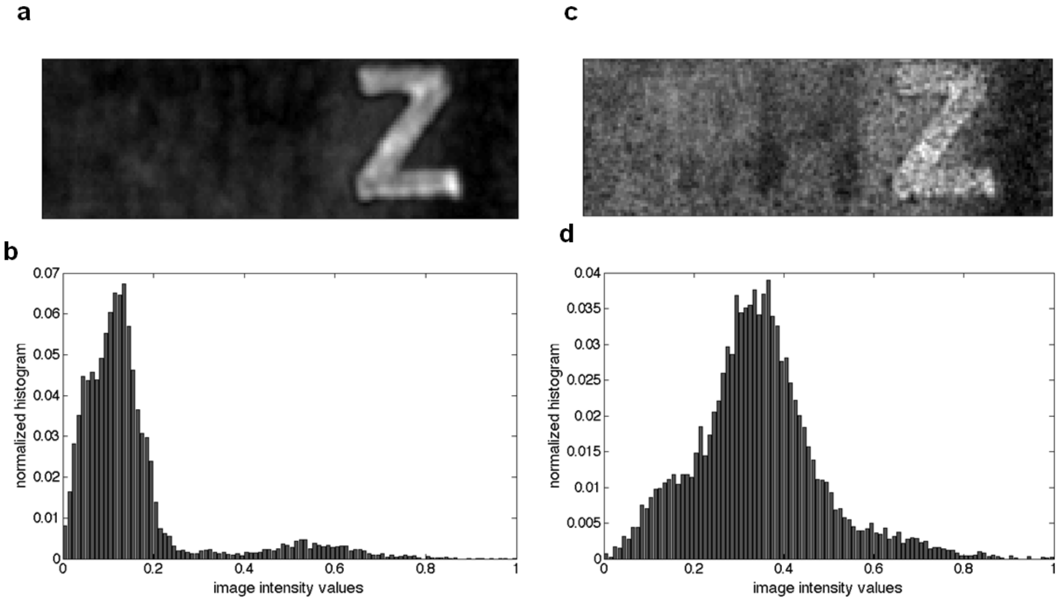

Supplementary figure 9 | Selection of the high contrast images based on the kurtosis value. a, A frequency image with relatively higher contrast. **b**, The histogram of the image in panel a, the kurtosis of the normalized image is 8.62. **c**, A relatively lower contrast frequency image. **d**, The histogram of the image in panel c which is more diffused than the histogram of image a; the kurtosis of the normalized image is 4.26.

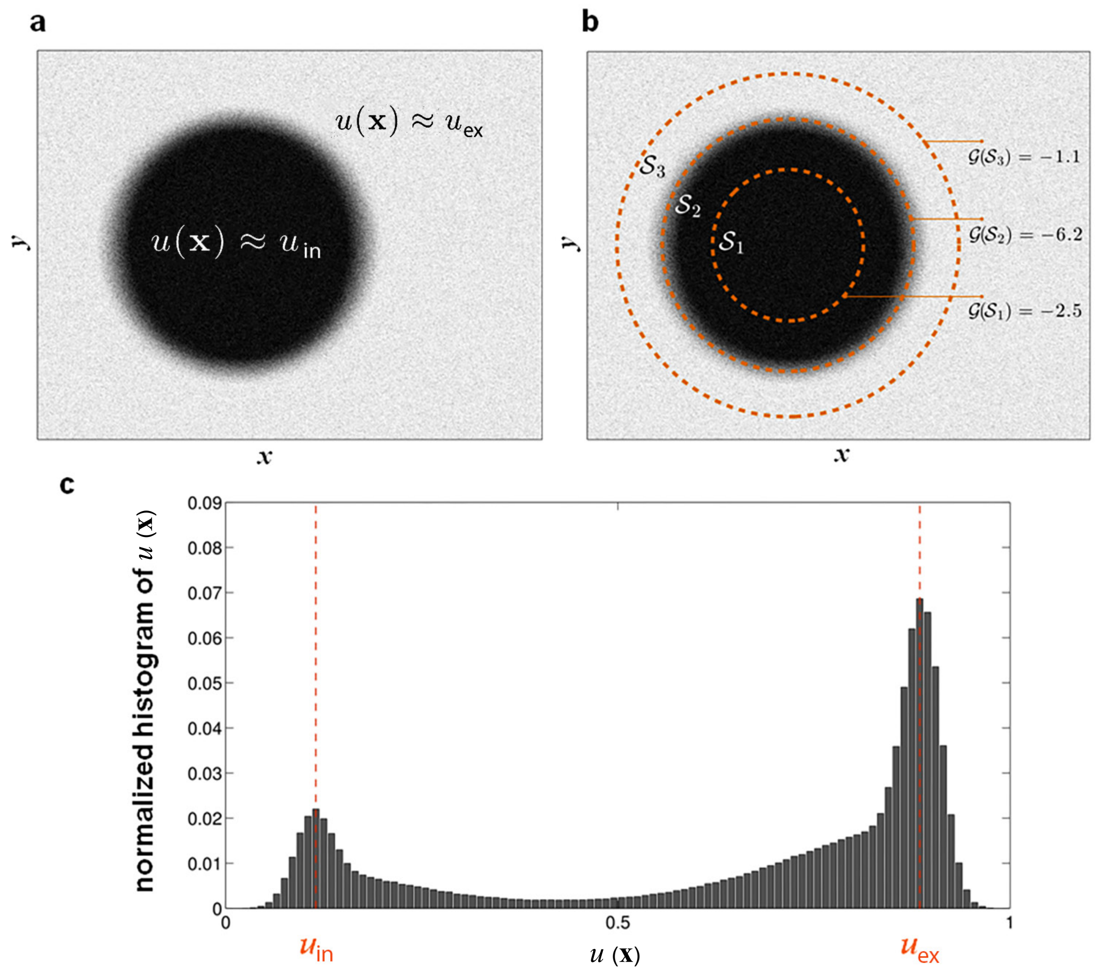

Supplementary figure 10 | Binary image segmentation. a, An almost binary image. **b**, Three disks of different radii and the corresponding cost values. **c**, Normalized histogram of the image and the corresponding values u_{in} and u_{ex}.

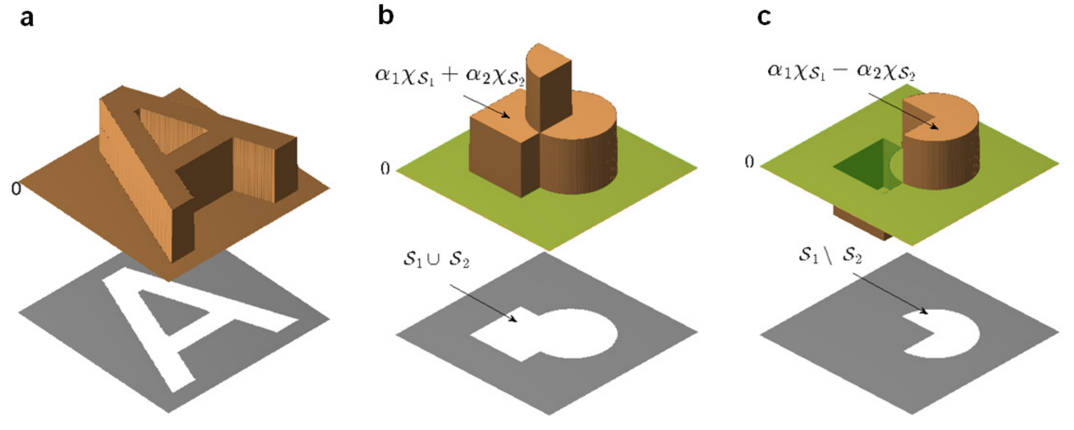

Supplementary figure 11 | Explanation of shape characteristic functions. **a**, An image (the character "A") and the corresponding characteristic function. **b**, For two given shapes S_1 and S_2, the positive support of $\alpha_1\chi_{S_1}(\mathbf{x}) + \alpha_2\chi_{S_2}(\mathbf{x})$ is $S_1 \cup S_2$ when $\alpha_1, \alpha_2 > 0$. **c**, The positive support of $\alpha_1\chi_{S_1}(\mathbf{x}) - \alpha_2\chi_{S_2}(\mathbf{x})$ is $S_1 \setminus S_2$ when $\alpha_2 > \alpha_1 > 0$.

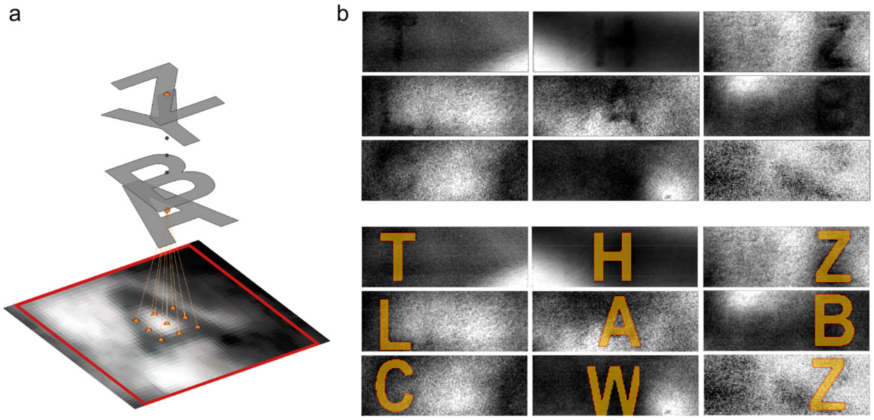

Supplementary figure 12 |Applying CCSC algorithm. a, The character placement in the dictionary: a stack of 26 characters is placed at each pivot point. **b**, The convex cardinal shape composition algorithm successfully extracts the characters down to page 7 for blue pen ink on polyethylene. Double-sided pages require the page contents to be flipped every other page in order to keep the dictionary intact.

Supplementary Tables

Supplementary Table 1 | Selected materials reflection and transmission properties. Values are measured from the amplitude of the time-domain waveform. Values can be considered as the values at peak frequency in the frequency-domain.

Layer Material	Content material	Reflectivity	Contrast vs layer	Transmission
300 μm paper	None	0.08 ± 0.02	--	0.72 ± 0.01
300 μm paper	Blue pen	0.10 ± 0.02	25%	0.71 ± 0.01
300 μm paper	6B pencil	0.15 ± 0.02	87%	0.68 ± 0.01
300 μm paper	HB pencil	0.10 ± 0.02	25%	0.71 ± 0.01
180 μm glossy paper	None	0.28 ± 0.02	--	0.85 ± 0.01
180 μm glossy paper	Blue pen	0.37 ± 0.02	32%	0.86 ± 0.01
180 μm glossy paper	Permanent ink	0.29 ± 0.05	3%	0.86 ± 0.01
180 μm glossy paper	HB pencil	0.28 ± 0.02	0%	0.79 ± 0.01
180 μm glossy paper	Laser printer ink	0.28 ± 0.02	0%	0.80 ± 0.01
190 μm polyethylene	None	0.22 ± 0.02	--	0.75 ± 0.01
190 μm polyethylene	Blue pen	0.24 ± 0.02	10%	0.66 ± 0.01

Supplementary Table 2 | Comparison of GPR vs. THz TDS

Measurement geometry	Frequency and metric ranges	Material properties focus	Inversion techniques	Layer content decomposition	2D context extraction
**GPR**	reflective and diffractive	narrow band complex permittivity	time domain filtering and deconvolution	Not applicable	Not applicable
**THz TDS**	reflective Confocal/raster	broadband absorption	time domain energy statistics	spectral kurtosis	convex cardinal

Supplementary Notes

Supplementary Note 1- Governing physics

Consider a dielectric slab of width d and reflection coefficient ρ, located perpendicular to the z axis in front of our system. When a linearly polarized electric field $\mathbf{E}^{+} = p(t - z/c)\hat{\mathbf{a}}_{\mathrm{x}}$ travels through the dielectric medium, a portion of the electric field is reflected back, which is in the form of $\mathbf{E}^{-} = r(t + z/c)\hat{\mathbf{a}}_{\mathrm{x}}$. The returned waveform $r(t)$ can be analytically calculated by convolving $p(t)$ with an impulse train, $s(t)$. Each impulse term in $s(t)$ corresponds to an inter-reflection within the slab. The spacing between the impulse spikes are $\tau = 2nd/c$, where n is the refractive index of the slab (that can be frequency dependant) and c is the wave speed in the vacuum. Based on the closed form expression[3] the first impulse is proportional to ρ, the second impulse weight is proportional to $\rho(\rho^2 - 1)$ and the subsequent impulse weights are proportional to $\rho^{2m-1}(\rho^2 - 1)$, for $m = 2, 3, \cdots$.

When ρ is small, only the first and second impulse terms in $s(t)$ are dominant and the remaining terms exponentially tend to zero. The location of these two terms corresponds to the front and back interfaces of the dielectric slab. Especially, when the pulse width is sufficiently small relative to the slab thickness, the two dominant peaks in the returned signal can identify the impulse locations and technically locate the dielectric boundaries. As an example, for $d = 300\ \mu$m, which is the paper thickness in our experiment, Supplementary figure 1 shows the simulated returned signal for the actual bipolar reference signal used in our experiments. We can observe that the peak locations exactly identify the impulse locations (shown in red), simply because the pulse effective width is small enough and the thickness of the paper is sufficiently large.

In the case of a multi-layer dielectric slab, the impulse response $s(t)$ effectively consists of multiple impulse pairs each corresponding to a layer, and under similar assumptions about the pulse width, the interface locations can be determined from the dominant peaks. Our Probabilistic Pulse EXtraction (PPEX) approach is in fact exploiting this fact to locate the paper boundaries, by identifying the peaks and following a statistical framework for possible model inaccuracies. If the effective pulse width is not sufficiently small, PPEX fails to locate the paper boundaries accurately. For example, PPEX cannot explicitly resolve the two reflections from the two boundaries of paper-air-paper as two peaks and it detects it as one peak. This is because these two peaks are only 20 μm (~0.06 ps) apart which is almost an order of magnitude smaller than the bandwidth of the pulse itself (2 THz or 150 μm) and fundamentally not resolvable with the system. This, however, does not interfere with the detection of the pages as the peaks from air-paper-air boundaries for each page are far enough to identify the length of time-gating window required for the next steps of the procedure. Additionally, even if an error were to occur for one of the pages, the spectral time-gating step would still increase the contrast of the content based on the average estimated time window, and the final convex cardinal shape decomposition would still overcome possible occlusions and distortions of the content. Therefore, the content extraction would not be directly affected. However it must be mentioned that, similar to any other THz-TDS system our system is fundamentally limited in depth resolution by half of its coherency length (~75 μm), therefore, if the pages where extremely thin (e.g. less than 75 μm) PPEX would repeatedly fail and estimate every two or three

boundaries as one. In this case, the negative results would be lower contrast enhancement in the spectral domain. Also, while the convex cardinal shape decomposition would still recover the content, there would be ambiguity as to which page (among few neighboring pages) these contents belong to.

Supplementary Note 2- Probabilistic Pulse Extraction (PPEX) approach and implementation

The extraction of the locations of pulses in a waveform is often realized through deconvolution and/or peak finding methods. Peak finding methods are used when overlapping between peaks is minimal or none. Deconvolution is used to mitigate the effects of the pulse width in cases in which the overlapping between pulses is significant.

PPEX computes the probability of each point being and extremal value in the time waveform. This is based on the amplitude, first derivative (e.g. velocity), and the statistical characteristics of the noise in the waveform. Ideally, extremal values are characterized because they are local maxima/minima and their derivative is equal to zero. PPEX starts by computing the time derivative of the waveform, or velocity. Then, both the amplitude and velocity are normalized with respect to their standard deviations:

$$(y,t) \rightarrow (q,p) = \left(y, \frac{dy}{dt}\right) \rightarrow (u,v) = \left(\frac{q}{\sigma_q}, \frac{p}{\sigma_p}\right). \qquad (1)$$

An energy value is defined for each point in the waveform based on amplitude and velocity. This energy is defined such that it is high for higher amplitudes and low velocities. This provides a filtering mechanism to separate signal from noise, retaining only the points that are candidates to be extremal:

$$E = u^2 e^{-v^2}. \qquad (2)$$

It is noteworthy that the optimal energy can be determined through a learning process, where a parametric model is learned using labeled peaks. Aside from the amplitude and the velocity, other features such as the peak width, spacing between the neighboring peaks and their location can be reflected in more complex energy models. For the purpose of this work a model solely in terms of u and v performed a sufficiently accurate characterization of the peaks.

The histogram for the amplitude and velocity of a waveform provides a statistical description of the distribution of their values. Considering that pulses in each terahertz waveform are highly localized in time, we can assume that most of the content of the waveform is noise and, thus, the histograms will mostly describe the statistical characteristics of the noise. In this case, the peaks of the pulses will tend to be outliers in the histogram of amplitudes but will lie around the zero value in the histogram of velocities. Histograms of experimental waveforms indicate that we could assume a Gaussian distribution for both the amplitudes and velocities. In this case, we can compute the probability of a point being an extremal point by using the error function for both the amplitude and velocity. Therefore, we can compute the likelihood of a point being an extremal point as:

$$p(u,v) \rightarrow 4\left(\mathrm{erf}(|u|) - \frac{1}{2}\right)\left(\frac{3}{2} - \mathrm{erf}(|v|)\right), \qquad (3)$$

where erf is the single sided cumulative error function, and u and v are the normalized amplitudes and velocities. This probability calculation provides a second threshold mechanism to select candidates with the highest likelihood of being extremal values. The result of applying PPEX on a waveform is a series of candidates that are likely to be extremal values. However, PPEX does not identify which candidate corresponds to a certain peak. Experimental and simulation results indicate that candidates tend to group around a real peak of the pulse. We use k-means clustering to group the candidates into the different peaks. The candidates have high likelihood to be the extremum; PPEX chooses the point with highest absolute amplitude inside each cluster as the representation of that peak. To enhance the performance of PPEX for very noisy data, a partial wavelet denoisng[4] can be considered as a preprocessing stage. Supplementary figure 1 shows the entire data processing flow.

The application of PPEX on the (x, y, z) THz time-domain data cube will provide candidates for extremal values of the temporal waveforms for each (x, y) pixel in the form of a point cloud. These extremal candidates provide the position of the different layers. Clustering will assign each candidate point into each layer so that a surface representing the layer can be fitted on. An alternative method to recover the position of the pages is to apply edge detection methods on the images representing the cross section of the sample, for example, using Canny edge detection[5,6] which is considered one of the most robust edge detection methods. Once the position of each page is determined, the intensity of the pulse can be mapped to generate an image of the intensity distribution across the layer. We use the PPEX output as an input to the time-gated spectral analysis.

Supplementary Note 3- PPEX comparison with conventional methods

Two major deconvolution approaches are used in the analysis of THz waveforms: frequency-based deconvolution and "CLEAN" deconvolution. Both methods require the measurement of reference pulse. In the frequency-based deconvolution approach, the response of the system $r(t)$ is modeled as the convolution of the reference pulse $p(t)$ with the response of the sample $s(t)$, which will contain the positions of the different layers modeled as a comb of impulse functions[7]. The position of the peaks is retrieved by Fourier inverting the division between the Fourier transform of the measured response and the Fourier transform of the reference pulse:

$$r(t) = p(t) * s(t) \rightarrow s(t) = FT^{-1}\left(\frac{\hat{R}(v)}{\hat{P}(v)}\right). \quad (4)$$

In CLEAN deconvolution, the reference pulse is shifted to where the first maximum or minimum peak is found in the waveform. The reference pulse is scaled and subtracted from the waveform. The resulting waveform may have another pulse in a different location. The process continues by shifting the reference pulse to the location of the new maximum or minimum and subtracting a scaled version from the waveform. This process is repeated recursively until a certain number of shifts or the noise floor is reached. The shifts indicate the positions of the peaks within the waveform[8]. CLEAN deconvolution operates entirely in the time-domain. Peak finding has been extensively studied and many review papers exist in the application specific and general context[9]. However, both peak finding and deconvolution do

not perform well in the presence of noise and low SNR conditions although some recent papers have proposed efficient automated peak finding algorithms in noisy data[2] .

To compare the performance of PPEX with different deconvolution and peak finding methods, we have used a Gaussian pulse of width equivalent to that of our reference pulse in the experimental measurements. We model the reflection of two interfaces with the reflection coefficient equivalent to that of the paper. The separation between pulses can be adjusted to simulate different overlapping conditions. To further evaluate the performance of the different methods we have also computed the global mean-square-error (MSE) in locating the position of the two peaks versus SNR. Intuitively, the log(MSE) for any algorithm decreases as the SNR increases until the slope of the curve asymptotes to that of the Cramer Rao lower bound[10,11]. Supplementary figure 3 shows the MSE for PPEX, CLEAN, frequency base deconvolution as reported in[7,8] and recent peak finding algorithm reported in[2,9]. The results of the simulation indicate that peak finding method starts to break down when SNR < 20 dB whereas frequency based deconvolution breaks down around 15 dB. Both CLEAN and PPEX perform better than frequency-based deconvolution or peak finding. However, PPEX breaks down around 5 dB whereas CLEAN starts failing around 8 dB. Therefore, PPEX has an edge over CLEAN in low SNR conditions. Our algorithm has the above distinguishability with regards to the noise variance and peak full width half maximum (FWHM), therefore in case of our THz measured data, peaks of ~450 fs separated by 400 fs can be distinguished from signal with SNR level as low as 8 dB. This is yet above the coherency limit of 250 fs.

The performance of PPEX versus Canny in extracting the position of a layer in cross section images is also simulated. The layer is simulated by shifting a Gaussian pulse along a tilted direction in the cross section and Gaussian white noise is added to the ground truth (Supplementary figure 4a). Due to the shape of the peak and the nature of edge detection, the results from applying Canny edge detection will provide the boundaries where the peak can be found but not the actual position of the peak (Supplementary figure 4b). On the other hand, PPEX shows candidate positions that are likely to be the peak instead of the edges (Supplementary figure 4c). The error is defined as the number of points detected as edge outside the FWHM of the original peak divided by the total number of points detected as edge in the image (Supplementary figure 4d). The results of the simulations indicate that PPEX is more robust and consistent in finding candidate peak positions compared to Canny as the SNR decreases. The simulations indicate that Canny begins to break down around 15 dB whereas PPEX breaks down around 3 dB (Supplementary figure 4e). The error is computed by averaging the output of simulating 100 trials for each SNR.

Supplementary figure 5 shows the results of applying CLEAN, Canny and PPEX on an experimental cross section (Supplementary figure 5a). The results indicate that PPEX is capable to retrieve the position of the layers more robustly than either CLEAN or Canny edge detection. These experimental results validate the simulations reported above.

Supplementary Note 4- PPEX versus wavelet-based peak finding

While wavelet transforms are dominantly used for filtering and denoising of a signal, they can also be used for peak finding. Supplementary figure 6 demonstrates the emitted signal waveform in time and Fourier domain along with a typical outcome of the PPEX compared against two wavelet-based peak finding techniques proposed in[1] and[2]. Wavelets can play two major roles in signal peak characterization.

In some techniques[1] wavelets mainly contribute in denoising the signal and a standard peak finding algorithm is applied to the processed data. Another approach is to use wavelets to decompose the signal into different scales. By inspecting the wavelet coefficients at a suitable scale, the peaks can be identified through the wavelets with the highest coefficients[2,12]. This approach may fail to produce promising results for noisy signals where the peaks have different widths or varying spacing.

Supplementary figure 6c shows the PPEX response to a sample signal in our experiments. Green circles mark correct identifications, whereas yellow and red circles mark missing or false identifications. In order to locate exact extrema, a local search around the outcomes of the k-means is performed. Supplementary figure 6d1 shows the performance of undecimated discrete wavelet transform[1] for the same level of smoothing used for PPEX. A higher smoothing destroys some of the major peak information as depicted in Supplementary figure 6d2. We have also demonstrated the identified peaks using the continuous wavelet-based pattern matching[2] for a small threshold (Supplementary figure 6e1) and a manually tuned threshold (Supplementary figure 6e2).

As indicated in this comparison while wavelet-based denoising can contribute to a statistical peak finding process (such as PPEX), using the wavelet transforms as the sole tool for peak finding can suffer from the distortion (varying width) and overlapping of the peaks in THz signals from densely layered structures. These overlappings and distortions are directly related to the thickness and complex THz permittivity of the layers and they affect wavelet-based methods more than PPEX since wavelet coefficient are notably affected with such signal distortions. Such sensitivity is appreciated in compression, filtering, and denoising applications but it's not directly of substantial benefit in peak detection. This may justify use of wavelet-based deconvolution discussed below, since deconvolution techniques are usually more robust to overlapping.

Supplementary Note 5- Wavelet-based deconvolution techniques

Using wavelets for the purpose of peak finding in THz time domain signals was discussed and analyzed in the previous section. We also discussed the frequency-based and CLEAN deconvolution techniques and showed their rather poor performance for our problem. The use of wavelets for the analysis of THz signals has shown promise, especially for deconvolution purposes[13–15]. A favorable property of the wavelets is the zero-mean nature of the scaling functions and waveform compatibility with THz signals. In this section we discuss two main deconvolution techniques equipped with wavelets to deconvolve the multilayer structure from the returned signal.

A promising technique in this area is proposed in[14], which suggests a frequency wavelet domain deconvolution (FWDD). The authors stabilize the standard frequency deconvolution using Wiener filtering, and equip their scheme with stationary wavelet shrinkage to accurately recover the impulse spikes in $s(t)$.

An alternative approach is to perform the standard deconvolution in time domain, while restraining $s(t)$ to a subspace spanned by wavelet basis[16]. More specifically, our sampled observations are in the form of $\mathbf{r} = \mathbf{p} * \mathbf{s} + \mathbf{n}$, where $\mathbf{p}$ is the known reference pulse in vector form, $\mathbf{n}$ is the measurement noise vector and $\mathbf{s}$ is to be determined. The convolution as a linear operator can be written as $\mathbf{p} * \mathbf{s} = \mathbf{T}_{\mathrm{p}}\mathbf{s}$, where $\mathbf{T}_{\mathrm{p}}$ is a Toeplitz matrix constructed from $\mathbf{p}$. Consider $\hat{\mathbf{s}}$ to be a vector containing the wavelet coefficients of $\mathbf{s}$,

based on which we can write $\mathbf{s} = \mathbf{W}\hat{\mathbf{s}}$. Here $\mathbf{W}$ carries the wavelet basis as its columns. We can directly deconvolve the measurements for $\hat{\mathbf{s}}$, through the following regularized least squares problem:

$$\hat{\mathbf{s}} = \underset{\mathbf{z}}{\operatorname{argmin}} \ \|\mathbf{r} - \mathbf{T}_{\mathrm{p}}\mathbf{W}\mathbf{z}\| + \lambda\|\mathbf{z}\|_p \ . \quad (5)$$

Here, $\|\boldsymbol{z}\|_p$ norm denotes the ℓ_p norm of the vector $\mathbf{z}$ and λ is a penalty weight. The simplest penalty takes the Tikhonov form for $p = 2$. However, since the THz waveforms of interest have simple representations in the wavelet domain, the vector $\hat{\boldsymbol{s}}$ must be sparse and the ℓ_1 penalty (i.e., $p = 1$) is a more reasonable choice.

Supplementary figure 7 shows the result of applying the aforementioned deconvolution schemes to our THz data. Supplementary figure 7c shows the FWDD outcome, which fails to locate the spikes in $s(t)$. Supplementary figure 7d shows the time domain deconvolution using the Tikhonov regularization and Supplementary figure 7e demonstrates the time domain deconvolution using the ℓ_1 penalty. We can see that the ℓ_1 penalized deconvolution generates the best results as it uses the sparse prior in $\hat{\mathbf{s}}$.

Supplementary figure 8 shows the recovered images by deconvolving the raw experimental data in Supplementary Figure 5a. We observe that only the time domain deconvolution using sparse prior is able to extract the layered structure of the medium. Despite this, identifying the layers is yet a challenging task, especially for pages 5 and after.

While the suggested deconvolution schemes show promise in many THz applications, they fail to show a reasonable performance in our experiments. The main reason behind such performance is the dispersion in our layered structure. Basically, in a dispersive media, the convolution model that we stated earlier in Supplementary Note 1 is no more valid and the returned signal undergoes a more complex model. This is why the FWDD scheme completely fails to locate the impulse locations and the sparse time domain deconvolution identifies a group of spikes rather than individual spikes around each interface. In fact, a small level of dispersion can drastically affect the deconvolution results, since compensation of the model mismatch may require a solution, which is far from the true impulse response. Therefore, PPEX is a more reliable technique here, since it uses a statistical framework to determine the layer boundaries. The use of PPEX, however, requires working with sufficiently thick layers, despite the proposed deconvolution schemes, which at least in theory do not impose such restriction.
Finally, we would like to note that even if PPEX fails to successfully locate the layers, the CCSC scheme can still identify the main characters in presence of overlapping characters from neighboring layers. The reader is referred to[17,18] for challenging identification examples in noisy and overlapping cases.

Supplementary Note 6- Time-Gated Spectral Analysis Framework

Corresponding to each layer, we have a cube of data $d(x, y, z)$ where x and y are the spatial coordinates and z is the time (convertible to depth). In discrete form, z takes values of $z_1, z_2, \cdots, z_n$, where each z_i corresponds to a certain page depth. These depths are found by the PPEX algorithm that was explained in Supplementary Note 1. We use the amplitude of discrete Fourier transform (DFT) of $d(x, y, z)$ along the z coordinate with window size of ~3 ps (or equivalently the ~660 μm depth in paper) to get $\hat{d}(x, y, \omega)$

(window size depends on layer thickness and is derived from the average thickness of layers found from PPEX) and subsequently its modulus denoted by

$$f(x, y, \omega) = |\hat{d}(x, y, \omega)|. \qquad (6)$$

The 3 ps window size is derived from PPEX output to minimize distortion from other layers. Here, ω takes k distinct values $\omega_1, \cdots, \omega_k$ because of the discrete nature of the transform.

In order to get clear images of the letters within each layer, an effective way of selecting the frequency bins is necessary to contrast the spectral difference between the blank paper and paper with content. We use higher order statistics and specifically the kurtosis information to select the high contrast images. For high contrast images that are closer to being binary, one would expect a histogram with sharper peaks about the low and high intensity values. The kurtosis value is a measure of the peakedness of a probability density function, and selection of frequency images with the highest kurtosis would provide us with the images of highest contrast (Supplementary figure 9).

After calculating the kurtosis values for all the frames $f(x, y, \omega_1)$ through $f(x, y, \omega_k)$ as

$$K_i = Kurt[\, f(x, y, \omega_i)\,] \quad i = 1, \cdots, k, \qquad (7)$$

the algorithm selects the frequency frames with highest kurtosis values and averages them. Specifically, we choose m' indices with highest kurtosis values and average them to produce the final time-gated Fourier domain image $T_{\mathrm{g}}(x, y)$. Compared to averaging along all the frequency frames or using a single frequency frame, this method improves the signal to noise ratio and avoids unwanted noise from higher frequencies. In summary, the time-gated spectral analysis does the following:

- time-gate based on the depth value found from PPEX
- calculate $K_1 = Kurt[\, f(x, y, \omega_1)\,]$ through $K_k = Kurt[\, f(x, y, \omega_{\mathrm{k}})\,]$
- sort the $K_1, \cdots, K_k$ to descending order as $K_{p_1}, K_{p_2}, \cdots, K_{p_k}$
- calculate $T_{\mathrm{g}}(x, y)$ as the average of m' frames with highest kurtosis values as below

$$T_{\mathrm{g}}(x, y) = \frac{\sum_{i=1}^{m'} f(x, y, \omega_{p_i})}{m'} \qquad (8)$$

It must be noted that the DFT is only taken along the z coordinate or equivalently the time coordinate; therefore, the frequency values are directly correlated to the absorption lines of the content materials and the paper materials. The Kurtosis is applied to a vectorized version of $f(x, y, \omega_i)$, which is a 2D image for a given ω_i.

In our experiments the frequency separation between the frames is 25 GHz. The frequency resolution is inverse of the window size, so the wider the window in the time-domain, the higher the frequency resolution. For the Fourier transform, we used a 139-point DFT (Discrete Fourier Transform). A search for the high contrast images is performed between the first 20 low frequency images and then the top three images with the highest kurtosis are selected ($m' = 3$). A simple averaging among the selected images provides us with a representative image associated with each layer.

Supplementary Note 7- Applying Convex Cardinal Shape Composition (CCSC)

To extract the characters after partial occlusion and with heavy noise we use our recently developed CCSC. To explain how CCSC works for our experiment we use the following example. Consider an image $\mathfrak{D}$ with pixel values $u(\mathbf{x})$ (in a 2D case, $\mathbf{x} = (x, y) \in \mathfrak{D}$) as shown in Supplementary figure 10a. A classic imaging problem is the binary image segmentation (BIS), which corresponds to partitioning $\mathfrak{D}$ into two disjoint regions; the interior region Σ and the exterior $\mathfrak{D}\backslash\Sigma$ or Σ^{c}. The measure of similarity that we use in this discussion is the intensity values; we essentially presume that the pixels values inside and outside Σ concentrate around constant values u_{in} and u_{ex}, respectively. The essence of our object identification scheme is illustrated with a simple toy example. Consider three disks S_1, S_2, and S_3, the boundaries of which are shown by orange the dashed-lines in Supplementary figure 10b. Let's focus on the problem of identifying the disk that better represents the dark region in Supplementary figure 10a (a quick visual comparison suggests S_2 to be the solution). We can view this problem as a BIS, where the optimal partitioner Σ is restricted to be an element of the set $\mathfrak{D} = \{S_1, S_2, S_3\}$. In a general setting, we refer to $\mathfrak{D}$ as the *shape dictionary*.

The binary image segmentation (BIS) problem, can in turn be cast as an optimization problem in terms of Σ. In images of almost binary nature, where a bimodal histogram is expected for the intensity values, the quantities u_{in} and u_{ex} can simply be taken to be the intensities corresponding to the peak frequencies of each mode (supplementary figure 10c). For such fixed values u_{in} and u_{ex}, a well-known variational formulation inspired by the Chan-Vese model[19,20] is determining the optimal partition by solving:

$$\Sigma^{*} = \arg\min_{\Sigma \epsilon \{S_1, S_2, S_3\}} \int_{\Sigma} \Delta(\mathbf{x}) d\mathbf{x}, \qquad (9)$$

where

$$\Delta(\mathbf{x}) \triangleq (u(\mathbf{x}) - u_{\text{in}})^2 - (u(\mathbf{x}) - u_{\text{ex}})^2. \qquad (10)$$

The first term in $\Delta(\mathbf{x})$ motivates identification of the dictionary elements with the least inner-variations around u_{in}. The second term motivates elements with the least outer-variations around u_{ex} by maximizing the inner-variations around this quantity. Together the two terms form a "push-pull" composite cost that promotes the best matching element. Some typical values for $\mathcal{G}(S_i) = \int_{S_i} \Delta(\mathbf{x}) d\mathbf{x}$, $i = 1,2,3$, are presented in supplementary figure 10b, which affirm the optimality of S_2 with respect to the problem in Supplementary Equation (9).

For a dictionary of prototype shapes $\mathfrak{D} = \{S_1, S_2, \dots S_n\}$, a more general shape identification scheme[20] corresponds to the minimization:

$$\min_{I_{\oplus}, I_{\ominus}} \int_{\mathcal{R}_{I_{\oplus}, I_{\ominus}}} \Delta(\mathbf{x}) d\mathbf{x} \quad \text{s.t.:} \quad |I_{\oplus}| + |I_{\ominus}| \leq s, \qquad (11)$$

where $|.|$ denotes the cardinality of the underlying sets, $s \,\epsilon\, \mathbb{N}$ is the maximum desired cardinality and $\mathcal{R}_{I_{\oplus}, I_{\ominus}}$ is a non-redundant composition of the dictionary elements as

$$\mathcal{R}_{I_\oplus, I_\ominus} \triangleq \left(\cup_{j\epsilon I_\oplus} S_j\right)\backslash\left(\cup_{j\epsilon I_\ominus} S_j\right). \qquad (12)$$

The sets $I_\oplus$ and $I_\ominus$ index the shapes we are adding and removing, respectively, \ denotes relative complement. The main difference between the proposed scheme in Supplementary Equation (11) and the typical minimizations of the form in Supplementary Equation (9) is that instead of identifying a single shape, Supplementary Equation (11) allows identification of a composition of the dictionary elements formed by $\left(\cup_{j\epsilon I_\oplus} S_j\right)\backslash\left(\cup_{j\epsilon I_\ominus} S_j\right)$. The number of elements present in the composition is controlled by the quantity s. For instance, in the case of $s = 2$, the search domain consists of the null-set and all compositions of the form $S_j, S_j \cup S_{j'}$ and $S_j \setminus S_{j'}$, where $j, j' \epsilon \{1,2, \dots, n\}$ and $j \neq j'$.

The composition form in Supplementary Equation (12) is a flexible model that allows identification of objects that consist of overlapping elements or occluded portions[20,21]. For our character recognition problem, this model allows identification of multiple (possibly overlapping) characters within an image in presence of partial occlusion resulted from the scattering of neighboring layers. Unfortunately, exact minimization of Supplementary Equation (11) is a very hard combinatorial problem. It would require an exhaustive search among an exponentially large number of possibilities and is therefore computationally intractable. As a remedy, a convex relaxation to Supplementary Equation (11) is proposed[22], which not only benefits computational tractability, but also provides a similar (and under certain conditions identical) performance as Supplementary Equation (11).

For a given shape S, the corresponding characteristic function, denoted by $\chi_S(\mathbf{x})$, is a function that takes unit values over the points $\mathbf{x} \in S$ and vanishes elsewhere (Supplementary figure 11a). Construction of the proposed convex proxy mainly relies on the fact that basic set operations among given shapes can be modeled by superimposing the corresponding characteristic functions (Supplementary figure 11b-c)[20–22] Ultimately, the proposed convex relaxation is cast as the minimization

$$\min_\alpha \int_{\mathfrak{D}} max(\Delta(\mathbf{x})\mathcal{L}_\alpha, \Delta(\mathbf{x})^-)d\mathbf{x} \quad \text{s.t.} \quad \|\alpha\|_1 \leq \tau, \qquad (13)$$

where $\mathfrak{D}$ is the domain of imaging, $\mathcal{L}_\alpha(\mathbf{x}) = \sum_{j=1}^{n} \alpha_j \chi_{S_j}(\mathbf{x})$, the ℓ_1 penalty $\|\alpha\|_1$ is simply $\sum_{j=1}^{n}|\alpha_j|$, and the quantity Δ^- takes the value of Δ when $\Delta < 0$ and vanishes otherwise.

Roughly speaking, in relating the minimizer of Supplementary Equation (13) to the optimal index sets associated with Supplementary Equation (11), the active α_j values identify the active shapes in the composition and their sign determines the index set ($I_\oplus$ or $I_\ominus$) they belong to. Inspired by ideas from sparse recovery[23], the ℓ_1 constraint is used to control the number of active shapes in the final representation and plays a similar role as s. An interesting property of the convex proxy Supplementary Equation (13) is that τ often takes integer values, and as elaborated in supplementary reference 13, for many problems of interest it is simply identical to s.

To apply the CCSC algorithm to our character extraction problem, we use a dictionary of English characters to match the letter in each layer with an element of the dictionary. Since each layer contains a single character (either placed on the left, center or the right portion of the image) to reduce the unnecessary computational load, the character recognition is performed in a loose window placed about the possible location of the character (Supplementary figure 12a). The possible loose *x-y* window is easily

localized based on signal level at the time-gated spectral image. To build up the shape dictionary, we place stacks of 26 uppercase letters at 9 different pivot points within the designated window. The pivot points are placed around the center of the window and at different locations as shown in supplementary figure 12a.

Throughout the experiments, the values u_{in} and u_{ex} are simply taken to be the 15% and 85% quantiles of the intensity values within each layer. Even such rough estimates of the mean texture values seem sufficient for a successful recovery of the characters. To eliminate the low frequency artifacts present in deep layers (layers 6 to 9), a slight filtering is performed to generate more homogenous images. Performing the convex scheme Supplementary Equation (13) on each image with $\tau = 1$, extracts the corresponding character. The character extraction results are provided in Fig. 3 of the manuscript for the paper and Supplementary figure 12b for plastic. In the case of plastic, we deal with a more challenging problem as the images are noisier and the contrast between layer and ink is smaller (e.g. blue pen over plastic reflectivity difference is 10% versus 87% or 6B over paper). Despite this, the CCSC algorithm is able to extract the correct characters down to layer 7, where the characters are yet visually recognizable. For layers 8 and 9 that almost no inference about the underlying character could be made, the identified letters are different than the true characters.

Supplementary Note 8- Material Contrast analysis at THz

The contrast between the reflectivity and transmission of different materials used for paper and written content is the ultimate bottleneck for extracting content from deeper layers. The reflection magnitudes are found by measuring the amplitude of the waveform in time-domain, which can be considered as representative frequency-domain value at peak frequency. Unlike Fresnel equations the measured reflectivity is affected by absorption of the material as well. Supplementary table 1 shows the THz contrast for a more extended set of different graphite-based pencils and pen inks over different substrate materials such as different paper types and plastic. The highest contrast is found for 6B pencil over the 300 μm-thick drawing paper. The lowest contrast is found for glossy paper with HB or laser printer ink. In all of our work we consider content layer or ink layer to be much thinner (about two orders of magnitude thinner) than the layer itself. Contrast is defined as the ratio of reflectivity difference of content plus layer divided by reflectivity of the layer material. Surely if one uses highly reflective inks (e.g. metallic ink) the contrast can exceed 100%.

While far in frequency and scale, reflection mode THz TDS and Ground Penetrating Radar (GPR) share similar essence in principle as they both send EM waves through layered structures. However, layer extraction in GPR is usually in the context of locating the dielectric depths after a very thick scattering layer based on refraction, diffraction, and amplitude modulation[24]. In other words the focus would be extracting the separating curves between the dielectric layers. That is why methods such as curvelet denoising and diffraction hyperbola matching have become dominant in this area of imaging[25]. Despite some level of similarity in principle, the focus of our work is extracting the depth surfaces and characterizing the contents within the extracted layers themselves. That is one of the main reasons why several advanced computational techniques needs to be cohesively developed in order to address our problem.

More specifically in the case of identifying the layer contents, in reading a closed book we encounter problems such as content contrasting, character overlapping, occlusion and basically shape interactions

which are not the type of problems encountered in GPR. This forces us to not only rely on the mutual time information in *x-y* but rather broad band spectral information at a fixed page. Our application heavily depends on the THz nature of the waves to exploit and characterize the spectral differences between different types of inks and papers. Table below further contrasts the two techniques.

Supplementary References:

1. Coombes, K. R. *et al.* Improved peak detection and quantification of mass spectrometry data acquired from surface-enhanced laser desorption and ionization by denoising spectra with the undecimated discrete wavelet transform. *Proteomics* **5,** 4107–17 (2005).
2. Du, P., Kibbe, W. A. & Lin, S. M. Improved peak detection in mass spectrum by incorporating continuous wavelet transform-based pattern matching. *Bioinformatics* **22,** 2059–2065 (2006).
3. Scharstein, R. W. Transient electromagnetic plane wave reflection from a dielectric slab. *IEEE Trans. Educ.* **35,** 170–175 (1992).
4. Donoho, D. L. De-noising by soft-thresholding. *IEEE Trans. Inf. Theory* **41,** 613–627 (1995).
5. Bao, P., Zhang, L. & Wu, X. Canny edge detection enhancement by scale multiplication. *IEEE Trans. Pattern Anal. Mach. Intell.* **27,** 1485–1490 (2005).
6. Canny, J. A computational approach to edge detection. *IEEE Trans. Pattern Anal. Mach. Intell.* **8,** 679–698 (1986).
7. Walker, G. C. *et al.* Terahertz deconvolution. *Opt. Express* **20,** 27230–27241 (2012).
8. Freedman, A., Bose, R. & Steinberg, B. D. Techniques to improve the CLEAN deconvolution algorithm. *J. Franklin Inst.* **332,** 535–553 (1995).
9. Zhang, J., Gonzalez, E., Hestilow, T., Haskins, W. & Huang, Y. Review of peak detection algorithms in liquid-chromatography-mass spectrometry. *Curr. Genomics* **10,** 388–401 (2009).
10. Bhandari, A., Kadambi, A. & Raskar, R. Sparse Linear Operator identification without sparse regularization? Applications to mixed pixel problem in Time-of-Flight/Range imaging. in *2014 IEEE Int. Conf. Acoust. Speech Signal Process.* 365–369 (IEEE, 2014).
11. Kay, S. M. *Fundamentals of Statistical Signal Processing*. (Volume I., Englewood Cliffs NJ Prentice Hall, 1993).
12. Cuiwei Li, Chongxun Zheng & Changfeng Tai. Detection of ECG characteristic points using wavelet transforms. *IEEE Trans. Biomed. Eng.* **42,** 21–28 (1995).
13. Parrott, E. P. J., Sy, S. M. Y., Blu, T., Wallace, V. P. & Pickwell-Macpherson, E. Terahertz pulsed imaging in vivo: measurements and processing methods. *J. Biomed. Opt.* **16,** 10601001–10601008 (2011).
14. Chen, Y., Huang, S. & Pickwell-MacPherson, E. Frequency-Wavelet Domain Deconvolution for terahertz reflection imaging and spectroscopy. *Opt. Express* **18,** 1177–90 (2010).
15. Chen, Y., Sun, Y. & Pickwell-Macpherson, E. Improving extraction of impulse response functions using stationary wavelet shrinkage in terahertz reflection imaging. *Fluct. Noise Lett.* **09,** 387–394 (2010).
16. Vonesch, C. & Unser, M. A fast thresholded landweber algorithm for wavelet-regularized multidimensional deconvolution. *IEEE Trans. Image Process.* **17,** 539–49 (2008).
17. Aghasi, A. & Romberg, J. Convex Cardinal Shape Composition and Object Recognition in Computer Vision. in *Asilomar Conf. Signals, Syst. Comput. Press*. 1541 - 1545 (IEEE, 2015).
18. Aghasi, A. & Romberg, J. Object Learning and Convex Cardinal Shape Composition. at <http://arxiv.org/abs/1602.07613>, (2016).
19. Chan, T. F. & Vese, L. a. Active contours without edges. *IEEE Trans. Image Process.* **10,** 266–277 (2001).
20. Aghasi, A. & Romberg, J. Sparse Shape Reconstruction. *SIAM J. Imaging Sci.* **6,** 2075–2108 (2013).
21. Aghasi, A., Kilmer, M. & Miller, E. L. Parametric Level Set Methods for Inverse Problems. *SIAM J. Imaging Sci.* **4,** 618–650 (2011).
22. Aghasi, A. & Romberg, J. Convex Cardinal Shape Composition. *SIAM J. Imaging Sci.* **8,** 2887–2950 (2015).
23. Romberg, J. Imaging via Compressive Sampling. *IEEE Signal Process. Mag.* **25,** 14–20 (2008).
24. Cassidy, N. J. *Ground Penetrating Radar Theory and Applications*. *Gr. Penetrating Radar Theory Appl.* (Elsevier, 2009).
25. Cieszczyk, S., Lawicki, T. & Miaskowski, A. The Curvelet Transform Application to the Analysis of Data Received from GPR Technique. *Electron. Electr. Eng.* **19,** 99–102 (2013).
